# On-Demand Acetaminophen Versus Scheduled Diclofenac-Serratiopeptidase After Cesarean Delivery: A Randomized Controlled Trial

**DOI:** 10.7759/cureus.114503

**Published:** 2026-08-13

**Authors:** Rajasri G Yaliwal, Devineni N Sree, Ambika H K., Shreedevi Kori

**Affiliations:** 1 Obstetrics and Gynaecology, Shri BM Patil Medical College, Vijayapura, IND

**Keywords:** acetaminophen, analgesia, cesarean delivery, diclofenac, postoperative pain

## Abstract

Background: Effective pain control after cesarean delivery supports maternal recovery, early mobilization, and newborn care. This study compared on-demand acetaminophen with scheduled diclofenac-serratiopeptidase for postoperative analgesia following cesarean delivery.

Materials and methods: This assessor-blinded randomized controlled trial included 400 women undergoing cesarean delivery at a tertiary-care teaching hospital in India. Participants were randomized equally into a study group and a control group. Both groups received injectable diclofenac during the first 48 postoperative hours. Thereafter, the study group received oral acetaminophen 500 mg on demand, while the control group received oral diclofenac 50 mg with serratiopeptidase 10 mg twice daily for five days. Pain was assessed using a 0-10 pain scale. Analgesic consumption and adverse effects were also recorded.

Results: The mean pain score at 48 hours was lower in the study group than in the control group (2.1±0.9 vs. 3.0±1.2; p<0.001). The study group also consumed fewer oral analgesic doses (4.2±1.5 vs. 5.8±2.0; p<0.001). A pain score below 4 was recorded in 176 (88%) participants in the study group and 154 (77%) in the control group (p=0.009). Gastric irritation was less frequent in the study group (3% vs. 10%; p=0.007), while nausea and vomiting were comparable.

Conclusion: On-demand acetaminophen was associated with lower oral analgesic consumption and less gastric irritation. Further studies using standardized treatment and assessment timings are needed.

## Introduction

Effective pain management in the post-cesarean period is critical for enhancing maternal recovery, promoting early mobilization, and improving overall patient satisfaction [1]. Traditionally, postoperative analgesia has followed standardized protocols involving the routine administration of nonsteroidal anti-inflammatory drugs (NSAIDs) or other analgesics at fixed intervals [2]. While this approach ensures consistent pain control, it may also lead to unnecessary medication exposure and potential side effects. An alternative strategy involves providing analgesics only when needed, allowing for individualized pain management that aligns more closely with the patient's actual pain experience [3].

The practice of administering analgesics on demand aims to reduce the risk of overmedication and minimize adverse effects while still achieving effective pain relief [4]. This approach empowers patients to communicate their pain levels and receive analgesia as required, potentially improving comfort and satisfaction [5]. Studies comparing routine scheduled analgesic regimens with on-demand strategies have yielded mixed results, with some indicating reduced medication use and fewer side effects in the on-demand groups, while others highlight concerns about inadequate pain control and delayed intervention when pain escalates [4,6].

In addition to pain management, the impact of analgesic regimens on wound healing is a critical consideration in the postoperative setting [7]. Excessive or inappropriate use of certain analgesics can negatively affect wound healing processes, either by impairing immune function or interfering with tissue regeneration [8]. Conversely, inadequate pain management can also hinder recovery by limiting mobility and contributing to stress, which may adversely affect wound healing [9]. Therefore, evaluating the balance between effective pain relief and optimal wound healing outcomes is essential when comparing different analgesic protocols [10].

The primary aim of this study is to optimize the dosage and use of analgesics in the post-cesarean period by comparing the requirement for analgesic drugs using an on-demand approach versus a standard protocol of routine NSAID administration. The study also provides insights into whether a more flexible, patient-driven approach to pain management could enhance recovery and reduce complications associated with routine medication use.

## Materials and methods

Study setting

This randomized controlled trial was conducted at the Department of Obstetrics and Gynaecology, Shri B.M. Patil Medical College Hospital and Research Centre, Vijayapura.

Study population

Consenting women aged ≥18 years undergoing cesarean section with no comorbidities were included in this study. Women with medical disorders, premature rupture of membranes (PROM), anemia, or abdominal edema were excluded.

Ethical considerations

Ethical approval was obtained from the Institutional Ethics Committee (reference no. BLDE(DU)/IEC/1091/2023-24) prior to initiation of the study. Written informed consent was obtained from all participants in accordance with the principles of the Declaration of Helsinki. The trial was prospectively registered with a national clinical trial registry of India (CTRI/2024/06/068542).

Sample size

The sample size was calculated based on the anticipated proportion of patients achieving a pain score <4 in the multimodal and traditional groups (77% and 88%, respectively) [3]. Using a two-sided p-value of 0.05, a power of 80%, and the formula n= [2p*q(zα+zβ)²]/MD², a total of 400 participants (200 per group) were required. Here, Z represents the Z statistic at the level of significance, MD denotes the anticipated difference between two proportions, P is the common proportion, and q is 100-p. This sample size was aimed at detecting a statistically significant difference in the efficacy of the two analgesic regimens in optimizing post-cesarean pain management.

Statistical analysis

Data were recorded in a structured proforma, including demographic details, clinical findings, analgesic use, and Visual Analog Scale (VAS) scores. Statistical analysis was performed using SPSS version 26 (Armonk, NY: IBM Corp.). Continuous variables were presented as mean±standard deviation (SD), and categorical variables as counts and percentages. The independent-samples t-test was used to compare normally distributed continuous variables between the two groups, while the Mann-Whitney U test was applied for non-normally distributed variables. Categorical variables were analyzed using the chi-square test or Fisher’s exact test, as appropriate. A two-sided p-value of <0.05 was considered statistically significant for all analyses.

Study design

The study was conducted as a single-blinded randomized controlled trial. Randomization was carried out using a computerized randomization chart generated through an online randomization platform. Participants were allocated to the intervention or control group based on this randomization sequence, while outcome assessment was performed without knowledge of group allocation to minimize observer bias.

Data collection

The study included 400 women undergoing cesarean sections who fulfilled the predefined inclusion criteria, with 200 participants in each group. Randomization was performed using a computer-generated random number table to allocate participants into the multimodal analgesia group or the traditional analgesia group. Written informed consent was obtained from all eligible participants prior to their inclusion in the study. A single-blinded study was conducted in which patients were blinded to which postoperative oral analgesic they would be given.

The enrolled participants were equally allocated into two groups as follows: group I (study group, multimodal analgesia): 200 mothers; group II (control group, traditional analgesia): 200 mothers.

In the multimodal analgesia group, patients received injectable Diclofenac AQ 75 mg for the first 48 hours postoperatively, followed by tablet acetaminophen 500 mg on a demand basis. Pain was assessed using the 10-cm VAS at the time of analgesic demand, with the total number of tablets consumed recorded at discharge. The VAS score ranged from 0 (no pain) to 10 (extreme pain), with scores categorized as none (0), mild (1-3), moderate (4-6), and severe (7-10). The traditional analgesia group received the same initial 48 hours of injectable Diclofenac AQ 75 mg, followed by a regimen of tablet diclofenac 50 mg combined with serratiopeptidase 10 mg for the subsequent five days. Pain assessment in this group was also conducted using the VAS, with comparable data collection methods.

If the patient’s pain did not subside even after administration of the analgesic assigned to the group, it was left to the discretion of the doctor to provide a rescue analgesic different from the one previously prescribed.

This study is a single-blinded study in which only the patient is unaware of which group they have been allocated to with respect to oral analgesics.

## Results

**Figure 1:**
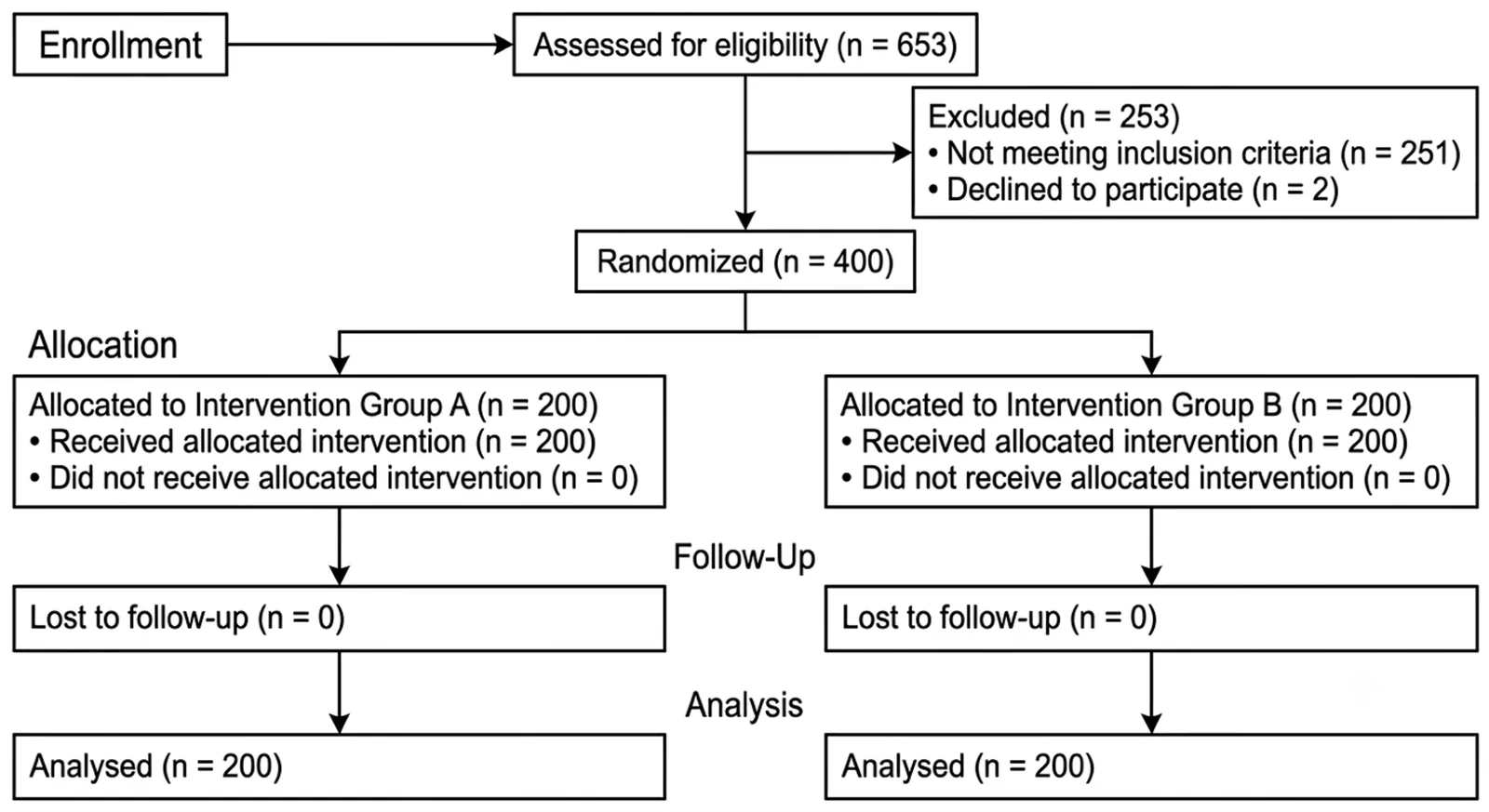
CONSORT flow diagram CONSORT, Consolidated Standards of Reporting Trials

A total of 653 women delivered during the study period (July 2024 to September 2024). Of these, 400 women were included in the study and randomized to either group I, receiving multimodal analgesia, or group II, receiving traditional analgesia (Figure 1).

The baseline demographic and clinical characteristics, including age, BMI (body mass index), parity, history of previous lower segment cesarean section (LSCS), and duration of surgery, were comparable between the two groups, with no statistically significant differences observed (p>0.05). The mean age of participants was 28.4±4.6 years in the study group and 29.1±4.8 years in the control group. The majority of participants in both groups were multiparous, and approximately 21% and 23% of participants in the study and control groups, respectively, had a history of previous LSCS (Table 1).

**Table 1:** Baseline demographic and clinical characteristics p<0.05 was considered statistically significant. BMI, body mass index; LSCS, lower segment cesarean section

Variable	Study group (n=200), mean±SD	Control group (mean±SD, n=200)	Test value	p-value
Age (years)	28.4±4.6	29.1±4.8	t=1.42	0.157
BMI (kg/m²)	26.7±3.2	27.1±3.4	t=1.18	0.238
Duration of surgery (minutes)	42.5±6.8	43.1±7.1	t=0.79	0.430
Variable	Study group (n=200), mean	Control group (mean±SD, n=200)	Test value	p-value
Parity (primipara/multipara)	90/110	85/115	χ²=0.25	0.617
Previous LSCS	42 (21%)	46 (23%)	χ²=0.20	0.655

The test used for the variables age, BMI, and duration of surgery was the independent unpaired Student's t-test. The test used for parity and previous LSCS was the chi-square test of independence.

Postoperative pain assessment using the VAS revealed significantly lower postoperative VAS scores in the study group compared to the control group at all measured intervals. At 48 hours after starting oral analgesics, the mean VAS score was 2.1±0.9 in the study group versus 3.0±1.2 in the control group (p<0.001). The total number of oral analgesic doses consumed by patients was significantly lower in the study group (4.2±1.5) compared to the control group (5.8±2.0, p<0.001). Additionally, a higher proportion of patients in the study group achieved a VAS score of less than 4 at 48 hours after starting oral analgesics (88% vs. 77%, p=0.009), demonstrating the superior efficacy of the multimodal analgesic regimen in pain control (Table 2).

**Table 2:** Postoperative pain and analgesic use p<0.05 was considered statistically significant. VAS, Visual Analog Scale

Variable	Study group (n=200)	Control group (n=200)	Test value	p-value
VAS score at 48 h after starting oral analgesics	2.1±0.9	3.0±1.2	t=7.01	<0.001
Total number of oral analgesic doses demanded	4.2±1.5	5.8±2.0	t=8.33	<0.001
Proportion of patients with VAS <4 at 48 h after starting oral analgesics	176 (88%)	154 (77%)	χ²=6.67	0.009

The tests used for determining the VAS score and total number of oral analgesic doses demanded were the independent unpaired Student's t-test. The test used to determine the proportion of patients with VAS less than 4 at 48 hours was the chi-square test of independence. Rescue analgesics were not provided to any patients.

Regarding adverse effects, the incidence of nausea, vomiting, and soft tissue healing delay was lower in the study group, although the differences were not statistically significant (p>0.05). However, the study group demonstrated a significantly reduced incidence of gastric irritation compared to the control group (3% vs. 10%, p=0.007). These findings suggest that the multimodal analgesic regimen not only provided better pain control but also had a more favorable safety profile with fewer gastrointestinal side effects (Table 3).

**Table 3:** Adverse effects of analgesic regimens p<0.05 was considered statistically significant. The statistical test used was the Pearson chi-square test.

Adverse effect	Study group (n=200)	Control group (n=200)	Test value	p-value
Nausea	12 (6%)	18 (9%)	χ²=1.09	0.297
Vomiting	8 (4%)	14 (7%)	χ²=1.75	0.185
Gastric irritation	6 (3%)	20 (10%)	χ²=7.28	0.007

## Discussion

Postoperative pain is a multifactorial process involving peripheral and central nociceptive pathways. Multimodal analgesia acknowledges this intricacy by employing diverse analgesic agents that target specific components of the pain pathway, thereby providing a more comprehensive approach to pain management. Non-opioid analgesics, such as NSAIDs and acetaminophen (paracetamol), are widely used as components of a multimodal regimen.

The findings from this randomized controlled trial underscore the efficacy of a multimodal analgesic regimen in optimizing postoperative pain management following cesarean delivery. The baseline demographic and clinical characteristics, including age, BMI, parity, history of previous LSCS, and duration of surgery, were comparable across both study groups, with no statistically significant differences observed (p>0.05).

The study revealed that patients in the multimodal analgesia group, who received injectable Diclofenac AQ for the initial 48 hours followed by acetaminophen on a demand basis, experienced significantly lower pain scores compared to those in the traditional analgesia group. Notably, the mean VAS score at 48 hours postoperatively was significantly lower in the multimodal group (2.1±0.9) than in the traditional group (3.0±1.2), with a p-value of <0.001. Acetaminophen functions by acting on the central nervous system (CNS) to diminish pain perception, while NSAIDs alleviate inflammation and inhibit pain signaling pathways. The combination of these agents facilitates more effective pain relief than the administration of a single agent alone. These results are consistent with a review study by Goel S et al., which posited that multimodal analgesia capitalizes on the strengths of multiple analgesic agents, each possessing distinct mechanisms of action [4].

Additionally, the total number of oral analgesic doses required was lower in the multimodal group (4.2±1.5) versus the traditional group (5.8±2.0, p<0.001). These findings suggest that an on-demand approach to pain management not only provides effective pain relief while reducing the overall consumption of analgesics but also minimizes unnecessary drug exposure and its associated risks. Reduced consumption of analgesics presents considerable benefits by minimizing potential side effects and drug-related complications, thereby making patient-driven pain management a more effective and safer approach [4].

These findings are consistent with previous research. Neall et al. also reported that multimodal analgesia strategies incorporating NSAIDs and acetaminophen resulted in improved postoperative pain scores and reduced opioid consumption compared to fixed-dose regimens [1].

Achieving a balance between adequate pain control and the avoidance of adverse effects is critical for ensuring patient safety and comfort. The study additionally evaluated the adverse effects associated with various analgesic regimens. While the incidence of nausea and vomiting did not exhibit significant differences between the groups, the multimodal group demonstrated a markedly lower incidence of gastric irritation (3% compared to 10%, p=0.007). This finding is particularly important, given that gastrointestinal side effects frequently pose concerns with the use of NSAIDs. Mitigating these adverse events may enhance patient compliance and satisfaction with pain management protocols. It is well documented that high doses or prolonged use of NSAIDs can lead to gastric irritation and, in certain instances, gastrointestinal ulcers [11].

By reducing the necessity for continuous NSAID administration and incorporating acetaminophen as an alternative, the multimodal regimen provides a safer option with fewer gastrointestinal complications.

The study aligns with results established by the updated PROSPECT recommendations for cesarean section, where postoperatively, analgesia should be patient- and procedure-specific, with a multimodal approach rather than a fixed schedule [12].

The findings of this study correlate with the ERAS (Enhanced Recovery After Surgery) recommendations, where the aim is to reduce pain postoperatively with minimal medical exposure. The factors studied here, such as reduced analgesic requirements and lower incidence of gastric irritation, support the principles of ERAS [13].

Most of the patients adhered to the oral medication regimen and asked for medication as and when pain was present. The maximum allowed use of acetaminophen was 4000 mg per day, while diclofenac was capped at 150 mg per day.

Limitations

This study was performed at one tertiary-care center, where generalizability is limited due to different patient dynamics and different analgesia given postoperatively. Performance bias might have been introduced because a single-blinded study allows the investigator to know which patient is receiving which treatment drug. The scoring used for postoperative pain is VAS scoring, which is a patient-based score, meaning it is subjective and may be influenced by multiple external factors. Another limiting factor is that pain measurement was performed early postoperatively. Pain arising later or chronically, as well as the quality of breastfeeding or maternal relief, has not been studied. The exclusion criteria of this study included females with several comorbidities, which could be a confounding factor and affect how the drugs play a different role as analgesics. Although adverse effects were documented, other factors such as cost-effectiveness, full ambulation, and return to normalcy were not documented.

## Conclusions

This study presents substantial evidence supporting a multimodal, patient-driven approach to pain management following cesarean deliveries. The implementation of individualized and multimodal strategies is associated with expedited recovery, optimized utilization of healthcare resources, and improved patient satisfaction. This methodology not only mitigates pain intensity and reduces analgesic consumption but also diminishes the likelihood of gastrointestinal side effects, establishing it as a safer and more effective alternative to conventional fixed-dose regimens. The intent of the study was to reduce the use of diclofenac as a traditional analgesic so as to reduce its known harmful side effects. Future research endeavors should investigate the long-term implications of various analgesic strategies on maternal recovery, overall patient satisfaction, and the potential cost-effectiveness of patient-centric pain management approaches. Refining pain management protocols in light of these findings could facilitate the development of more effective and patient-centered healthcare practices in the fields of obstetrics and gynecology.
